# Associations of psychological safety and perceptions of organizational environment among healthcare workers: a cross-sectional study in a university hospital

**DOI:** 10.1186/s12913-026-15330-3

**Published:** 2026-08-07

**Authors:** Tsutomu Koike, Teruhiko Imamura, Kota Kakeshita, Koichiro Kinugawa

**Affiliations:** The Second Department of Internal Medicine, University of Toyama, 2630 Sugitani, Toyama, 930-0194 Japan

**Keywords:** Psychological safety, Staff satisfaction, Perceptions of their organizational environment, Healthcare workers

## Abstract

**Background:**

Psychological safety is increasingly recognized as an important organizational factor in healthcare, yet its relationship with staff satisfaction at the institutional level remains unclear. This study examined the association between psychological safety and staff satisfaction using perceptions of their organizational environment framework.

**Methods:**

We conducted a single-center cross-sectional survey of healthcare workers at a university hospital in November 2023. Staff satisfaction and psychological safety were assessed using a web-based anonymous questionnaire. The overall evaluation score was used as a global indicator of perceptions of their organizational environment. Importance–satisfaction analysis identified priority domains, and multivariable linear regression evaluated associations between psychological safety and outcomes.

**Results:**

Among 685 participants, retention intention and perceived quality of care were identified as priority improvement domains. All psychological safety items correlated positively with overall evaluation (*p* < 0.05). In multivariable analyses, “skills and talents valued and utilized” (β = 0.381, *p* < 0.001) and “able to bring up problems” (β = 0.188, *p* < 0.001) showed the strongest associations with organizational evaluation. Similar relationships were observed for priority domains. Employees in their 30s demonstrated lower scores than older groups.

**Conclusions:**

Psychological safety, particularly the speak-up climate and recognition of professional expertise, was strongly associated with healthcare workers’ overall perceptions of their organizational environment.

**Supplementary Information:**

The online version contains supplementary material available at https://doi.org/10.1186/s12913-026-15330-3.

## Introduction

In recent years, the healthcare environment in Japan has faced major challenges, including rapid population aging, increasing healthcare demands, and shortages of healthcare professionals. Under these circumstances, healthcare institutions are required to establish systems that enable the efficient and safe delivery of high-quality care. Improving healthcare quality depends not only on individual professional competence but also on organizational factors such as workplace environment and culture.

Perceptions of their organizational environment and workforce experience are increasingly recognized as determinants of healthcare system performance [1]. Previous studies have shown that higher staff satisfaction is associated with greater employee loyalty, improved service quality, and better patient experiences [2]. Accordingly, staff satisfaction is recognized as an important organizational indicator linked to healthcare quality. However, increasingly complex patient needs and evolving healthcare policies have made it difficult for many institutions to implement effective strategies to improve staff satisfaction [3]. Consequently, understanding healthcare workers’ perceptions of their organizational environment has become increasingly important.

The concept of “psychological safety” had been discussed in organizational research before Edmondson’s work. Edmondson subsequently conceptualized psychological safety at the team level as a shared belief that the team is safe for interpersonal risk-taking, such as speaking up or expressing concerns [4]. Growing evidence suggests that psychological safety plays a critical role in team-based healthcare and patient safety. Low psychological safety may increase psychological stress and reduce work motivation, whereas higher psychological safety promotes learning behaviors, incident reporting, and improved team performance [5–9]. These findings indicate that psychological safety may represent a key organizational factor shaping healthcare workers’ overall perception of their workplace.

Nevertheless, studies examining the relationship between psychological safety and staff satisfaction at the institutional level remain limited. In particular, few investigations have evaluated psychological safety within a framework that considers overall organizational evaluation as an integrated perception derived from multiple workplace domains. Whether psychological safety contributes to healthcare workers’ global perception of organizational performance remains unclear.

Our institution conducts an annual staff satisfaction survey to evaluate workplace experiences and organizational challenges. In this study, we incorporated measures of psychological safety into this survey to examine its association with overall organizational evaluation. We conceptualized the overall evaluation score as a global indicator of healthcare workers’ perceptions of their organizational environment and investigated how workplace factors, including psychological safety, contribute to this integrated assessment. Here, healthcare workers’ perceptions of their organizational environment refer to employees’ overall subjective assessment of their workplace, as measured by the global evaluation item. This study aims to provide foundational evidence suggesting that improvements in psychological safety may ultimately contribute to enhanced patient satisfaction through improved staff experience at the organizational level.

## Methods

### Study design and participants

This study was a single-center, questionnaire-based cross-sectional study conducted in accordance with the Strengthening the Reporting of Observational Studies in Epidemiology guidelines and Declaration of Helsinki. All full-time and part-time employees working at our institution, including physicians, nurses, pharmacists, allied health professionals, administrative staff, and other personnel, were eligible to participate.

An anonymous web-based survey assessing staff satisfaction and psychological safety was conducted over two weeks in November 2023, dominantly using Google Forms. Participation was voluntary, and several reminder notifications were distributed during the survey period to encourage participation. The survey was distributed to all 1,700 employees at the institution, and responses were obtained from 685 individuals (response rate: 40.3%), including 157 physicians, 257 nurses, 25 pharmacists, 99 allied health professionals, 105 administrative staff members, and 42 individuals in other roles.

No personally identifiable information was collected. The requirement for written informed consent was waived due to the anonymous nature of the survey. The study protocol was approved by the Institutional Ethics Committee of our institution (approval number: H2025056).

### Survey instruments

Survey items are summarized in Supplementary Table 1. Staff satisfaction was assessed using a standardized survey framework provided by the Japan Council for Quality Health Care (Supplementary Table 2). This is widely used in Japanese healthcare institutions as a standardized instrument for assessing multiple domains of staff satisfaction and organizational performance. The questionnaire consisted of 11 structured items, including an item corresponding to the overall evaluation. The overall evaluation item is intended to capture respondents’ global assessment of their workplace. Although the overall evaluation item has not been independently validated as a standalone measure, it is incorporated into the standardized survey framework and is intended to represent respondents’ overall assessment of the organization.

Psychological safety was assessed using the seven-item scale originally developed by Edmondson [4], administered using a validated Japanese-language version (Supplementary Tables 1, 2). This scale evaluates the extent to which team members feel safe to speak up, express concerns, and take interpersonal risks within their workplace environment.

All structured questionnaire items, including the 11 staff satisfaction items and the seven psychological safety items, were rated using a five-point Likert scale (1 = strongly disagree, 2 = disagree, 3 = neutral, 4 = agree, 5 = strongly agree). All questions were phrased positively so that higher scores indicated greater satisfaction or higher perceived psychological safety. Each item was analyzed individually and treated as a continuous variable.

### Outcome definition and study framework

The overall evaluation item was predefined as the primary outcome representing global healthcare workers’ perceptions of their organizational environment. This item was derived from the standardized staff satisfaction survey system developed by the Japan Council for Quality Health Care and reflects respondents’ overall assessment of the organization.

Individual staff satisfaction items and psychological safety items were treated as explanatory variables representing specific domains of workplace experience. The study examined the associations between these explanatory factors and the overall evaluation score. All questionnaire items were mandatory in the web-based survey; therefore, no item-level missing data were present.

### Statistical analyses

Continuous variables are presented as mean ± standard deviation, and categorical variables are summarized as counts and percentages. A two-sided p-value < 0.05 was considered statistically significant. All statistics were performed using JMP pro ver14.2 (SAS Institute Inc., Cary, NC, USA). All analytical procedures were predefined before outcome analyses to minimize exploratory bias.

Associations between the overall evaluation score and individual staff satisfaction items, as well as psychological safety items, were initially examined using Pearson’s correlation coefficients.

To identify organizational domains requiring improvement, an importance–satisfaction analysis was conducted. For each staff satisfaction item excluding the overall evaluation, mean satisfaction scores were calculated and plotted against their corresponding Pearson correlation coefficients with the overall evaluation score. In this framework, the mean satisfaction score represented the level of satisfaction, whereas the correlation coefficient with the overall evaluation was interpreted as an indicator of relative importance.

Items were subsequently mapped onto a two-dimensional matrix using the overall mean satisfaction score and the mean correlation coefficient as reference thresholds. The matrix was divided into four predefined domains: status quo maintenance (high satisfaction and low importance), future improvement (low satisfaction and low importance), priority to maintain (high satisfaction and high importance), and priority for improvement (low satisfaction and high importance). Items categorized as priority for improvement were considered key organizational targets for subsequent analyses. This analytical approach has been widely applied in organizational and service quality research to prioritize improvement strategies.

To evaluate independent associations between psychological safety and healthcare workers’ perceptions of their organizational environment, multivariable linear regression analyses were performed with the overall evaluation score as the dependent variable and the seven psychological safety items, as well as male sex, age (younger than 30 years) and job types (clinical staff vs. non-clinical staff) as explanatory variables. Before the multivariable regression analyses, multicollinearity among the explanatory variables was assessed using variance inflation factors, and we confirmed that all variables had variance inflation factors < 5.0 beforehand. Model fit was evaluated using R² values. Regression model assumptions were assessed by visual inspection of residual plots to evaluate linearity, homoscedasticity, and influential outliers.

Additional regression analyses were conducted using retention intention and perceived quality of care as dependent variables to examine whether psychological safety factors identified in the primary analysis demonstrated consistent associations across priority organizational domains.

Subgroup analyses according to age categories were performed for outcomes identified as priority domains. Multiple comparisons were adjusted using the Bonferroni method.

## Results

### Participant characteristics

Participant characteristics are summarized in Table 1. A total of 685 employees participated in the study, including 231 men (33.7%) and 454 women (66.3%). Nurses constituted the largest professional group, and participants in their 30s represented the most common age category.

**Table 1:** Background of subjects

Category	*N* (%)
Total participants	685
Male	231 (33.7%)
Female	454 (66.3%)
Medical doctors	157 (22.9%)
Nurses	257 (37.5%)
Pharmacists	25 (3.6%)
Allied health professionals	99 (14.5%)
Administrative staff	105 (15.3%)
Others	42 (6.1%)
Age 20–29 years	149 (21.8%)
Age 30–39 years	181 (26.4%)
Age 40–49 years	177 (25.8%)
Age 50–59 years	129 (18.8%)
Age 60–69 years	49 (7.2%)

Variables are presented as counts (percentages)

### Overall staff satisfaction and item-level associations

The results of the staff satisfaction survey are presented in Table 2. The mean score for the overall evaluation, representing global staff satisfaction, was 2.92 ± 1.14. Correlation analyses demonstrated that all individual satisfaction items were positively associated with the overall evaluation score. Among these, retention intention and perceived quality of care showed the strongest correlations with overall evaluation (*r* = 0.76 and *r* = 0.75, respectively) (Table 2).

**Table 2:** Staff satisfaction and psychological safety scores

Question	Mean ± SD	Correlation with overall evaluation (*r*)
***Employee Satisfaction***		
** Overall perceptions of their environment**	2.92 ± 1.14	**-**
Workplace atmosphere and relationships	3.72 ± 0.98	0.45*
Job satisfaction	3.82 ± 0.98	0.46*
Employment conditions	2.82 ± 1.22	0.51*
Working conditions	3.27 ± 1.24	0.44*
Learning and growth opportunities	3.77 ± 0.97	0.40*
Mental anxiety (reverse meaning)	2.84 ± 1.16	0.51*
Fair evaluation	3.12 ± 1.07	0.51*
Trust in supervisor	3.76 ± 1.07	0.45*
Retention intention	3.21 ± 1.14	0.76*
Perceived quality of care	3.23 ± 1.12	0.75*
***Psychological Safety***		
Mistakes not held against members	3.40 ± 1.06	0.33*
Able to bring up problems	3.43 ± 1.06	0.41*
No rejection for being different	3.55 ± 1.02	0.34*
Safe to take interpersonal risks	3.45 ± 1.04	0.35*
Easy to ask colleagues for help	3.89 ± 0.94	0.38*
No deliberate undermining of others’ efforts	3.92 ± 1.05	0.38*
Skills and talents valued and utilized	3.53 ± 0.96	0.48*

Continuous variables are presented as mean ± standard deviation. *p < 0.05 by Pearson’s correlation coefficient versus overall evaluation. SD, standard deviation

### Identification of priority domains

**Fig. 1:**
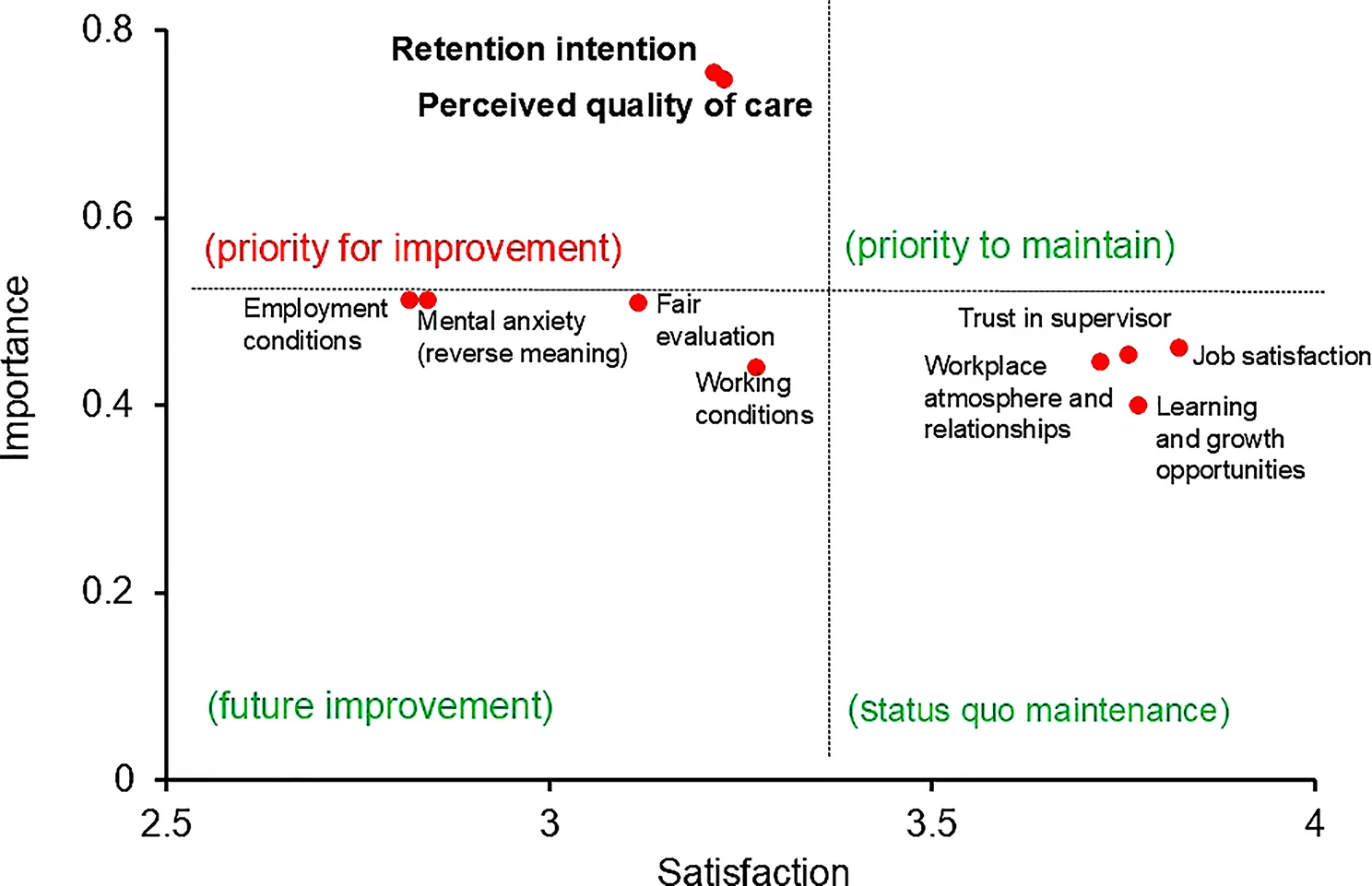
Importance–satisfaction matrix of staff satisfaction items. Importance–satisfaction matrix showing the relationship between mean satisfaction scores and their corresponding correlations with overall organizational evaluation for each staff satisfaction item. The horizontal axis represents mean satisfaction scores, and the vertical axis represents Pearson’s correlation coefficients with the overall evaluation score. Reference lines indicate the overall mean satisfaction score and mean correlation coefficient, dividing items into four domains: status quo maintenance, future improvement, priority to maintain, and priority for improvement. Retention intention and perceived quality of care were categorized within the priority for improvement domain

The importance–satisfaction analysis identified retention intention and perceived quality of care as the two domains requiring the highest priority for organizational improvement (Fig. 1). Both variables were located in the “priority for improvement” quadrant, indicating high importance but relatively low satisfaction levels.

### Association between psychological safety and perceptions of their organizational environment

Mean scores for psychological safety items ranged between 3.00 and 4.00 (Table 2). Each psychological safety item demonstrated a significant positive correlation with the overall evaluation score (*p* < 0.05 for all).

Among these items, “able to bring up problems” and “skills and talents valued and utilized” showed higher correlation coefficients with overall evaluation than the other psychological safety items.

### Multivariable analyses

In multivariable linear regression analyses using overall evaluation as the dependent variable and the seven psychological safety items, sex, age and job type as explanatory variables, the strongest independent association was observed for “skills and talents valued and utilized” (β = 0.377, *p* < 0.001), followed by “able to bring up problems” (β = 0.183, *p* < 0.001) (Table 3). In addition, the perception of “no deliberate undermining of others’ efforts” was also significantly associated with overall evaluation (β = 0.096, *p* = 0.046).

**Table 3:** Effects of each psychological safety item on the overall perceptions of their environment

Variables	β	t-value	*p*-value	95% CI
Mistakes not held against members	0.083	1.689	0.092	-0.013–0.180
Able to bring up problems	0.183	3.304	< 0.001*	0.074–0.291
No rejection for being different	0.000	0.006	0.995	-0.115–0.115
Safe to take interpersonal risks	-0.063	-1.015	0.311	-0.186–0.059
Easy to ask colleagues for help	0.089	1.613	0.107	-0.019–0.197
No deliberate undermining of others’ efforts	0.096	1.999	0.046*	0.002–0.190
Skills and talents valued and utilized	0.377	7.176	< 0.001*	0.274–0.481
Male sex	-0.022	-0.270	0.787	-0.182–0.138
Age (younger than 30 years)	-0.206	-2.689	0.007*	-0.357–-0.056
Job type (clinical staff vs. non-clinical staff)	-0.060	-0.650	0.516	-0.245–0.123

*p < 0.05 by multiple regression analyses for the overall evaluation. CI, confidence interval

Adjusted R² = 0.283

Further regression analyses were conducted using retention intention and perceived quality of care—identified as priority improvement domains—as dependent variables. Similar patterns were observed, with “able to bring up problems” and “skills and talents valued and utilized” being valued as significant explanatory factors for both outcomes, demonstrating consistent associations across priority organizational domains (Tables 4 and 5).

**Table 4:** Effects of each psychological safety item on the retention intention

Variables	β	t-value	*p*-value	95% CI
Mistakes not held against members	0.028	0.551	0.582	-0.071–0.126
Able to bring up problems	0.195	3.459	0.001*	0.084–0.305
No rejection for being different	-0.004	-0.081	0.935	-0.122–0.112
Safe to take interpersonal risks	-0.045	-0.712	0.477	-0.170–0.080
Easy to ask colleagues for help	0.027	0.490	0.625	-0.170–0.080
No deliberate undermining of others’ efforts	0.090	1.828	0.068	-0.007–0.186
Skills and talents valued and utilized	0.370	6.907	< 0.001*	0.265–0.476
Male sex	-0.047	-0.571	0.568	-0.210–0.115
Age (younger than 30 years)	-0.244	-3.121	0.002*	-0.398–-0.090
Job type (clinical staff vs. non-clinical staff)	-0.386	-4.042	< 0.001*	-0.573–-0.198

*p < 0.05 by multiple regression analyses for the retention intention. CI, confidence interval

Adjusted R² = 0.254

**Table 5:** Effects of each psychological safety item on the quality of care

Variables	β	t-value	*p*-value	95% CI
Mistakes not held against members	0.043	0.863	0.388	-0.055–0.141
Able to bring up problems	0.168	2.996	0.002*	0.058–0.278
No rejection for being different	0.057	0.956	0.340	-0.060–0.173
Safe to take interpersonal risks	-0.046	-0.721	0.471	-0.170–0.079
Easy to ask colleagues for help	0.063	1.123	0.262	-0.047–0.172
No deliberate undermining of others’ efforts	0.069	1.426	0.154	-0.026–0.165
Skills and talents valued and utilized	0.299	5.611	< 0.001*	0.194–0.414
Male sex	-0.051	-0.620	0.535	-0.213–0.111
Age (younger than 30 years)	-0.251	-3.226	0.001*	-0.404–-0.098
Job type (clinical staff vs. non-clinical staff)	-0.215	-2.269	0.024*	-0.401–-0.029

*p < 0.05 by multiple regression analyses for the quality of care. CI, confidence interval

Adjusted R² = 0.228

### Age-related differences in key outcomes

**Fig. 2:**
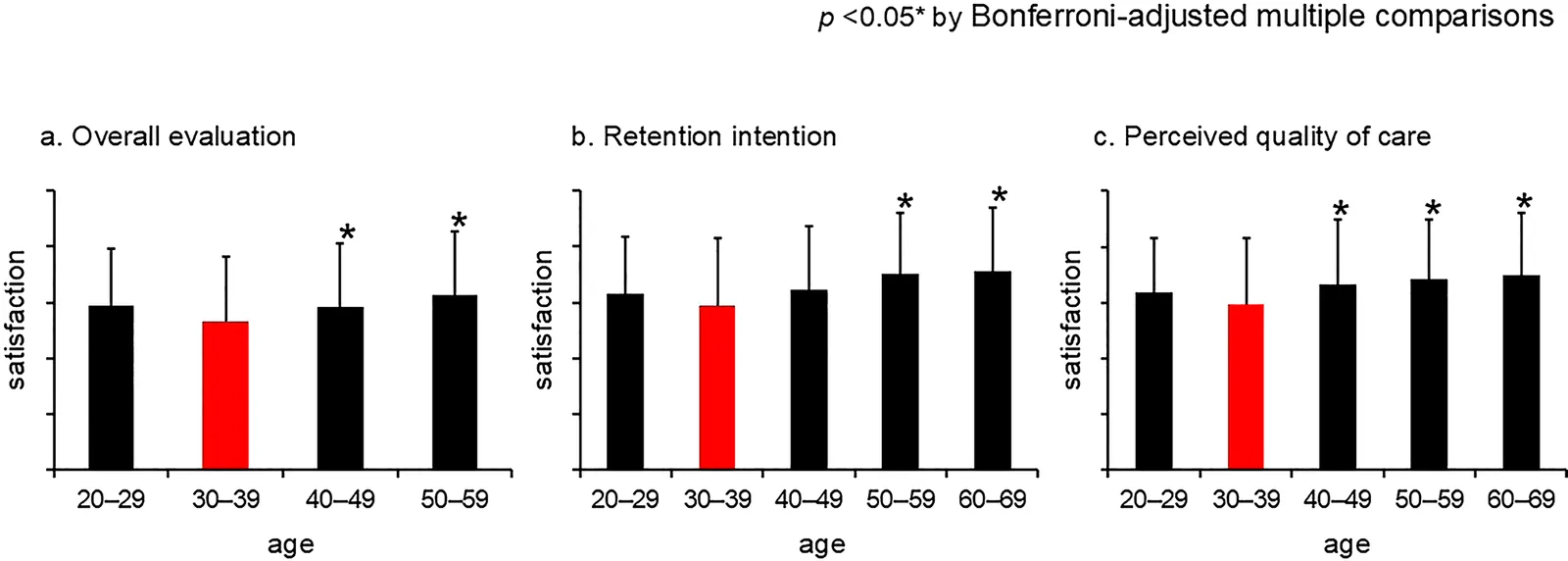
Age-stratified comparisons of key organizational outcomes. Age-stratified comparisons of (**a**) overall organizational evaluation, (**b**) retention intention, and (**c**) perceived quality of care. Data are presented as mean scores across age groups. Statistical comparisons were performed using Bonferroni-adjusted multiple comparisons. Employees in their 30s demonstrated significantly lower scores compared with older age groups (*p* < 0.05)

As an additional analysis, subgroup analyses according to age were performed. Age-stratified results for overall evaluation, retention intention, and perceived quality of care are shown in Fig. 2.

Bonferroni-adjusted multiple comparisons revealed that employees in their 30s had significantly lower overall evaluation and retention intention scores than those in their 50s and 60s (*p* < 0.05; Fig. 2a and b). Similarly, perceived quality of care scores were significantly lower among employees in their 30s compared with those in their 40s, 50s, and 60s (*p* < 0.05; Fig. 2c).

## Discussion

This study examined the association between psychological safety and healthcare workers’ perceptions of their organizational environment in a university hospital using the perceptions of their organizational environment framework. Two principal findings emerged. First, retention intention and perceived quality of care were identified as priority domains requiring organizational improvement. Second, among psychological safety components, the ability to speak up about workplace problems and the perception that one’s skills and talents are respected demonstrated the strongest and most consistent associations with overall organizational evaluation, as well as with both priority domains. These findings suggest that specific aspects of psychological safety are closely linked to healthcare workers’ global perception of their organizational environment. Although conducted in a single Japanese university hospital, the organizational mechanisms identified in this study—particularly the roles of psychological safety and speak-up climate—are likely applicable across healthcare systems facing workforce shortages and increasing care complexity.

### Retention intention and perceived quality of care as priority organizational issues

Retention intention and perceived quality of care emerged as key improvement targets, particularly among employees in their 30s, a group essential for both current clinical operations and future institutional sustainability. Previous studies in Japan report that a substantial proportion of healthcare workers consider leaving their workplace [10], and retention intention is a strong predictor of turnover behavior [11]. Workforce attrition may reduce clinical capacity and compromise healthcare delivery. Importantly, perceived quality of care represents not only an evaluation of clinical performance but also a reflection of how healthcare workers experience organizational functioning and teamwork within their workplace. Mid-career professionals often face increasing professional demands alongside life-stage challenges, which may contribute to declining organizational attachment and reduced perceptions of care quality within demanding clinical environments. Our findings suggest that retention intention among this group may warrant particular organizational attention.

### Psychological safety in academic medical centers

Academic medical centers operate under high-performance expectations because of their combined clinical, research, and educational responsibilities. Previous organizational research has suggested that psychological safety is essential for maintaining learning, communication, and employee well-being in such a demanding environment [12]. Although our study did not evaluate organizational zones proposed by Edmondson, our findings support the importance of psychological safety as an organizational factor associated with favorable workplace perception.

### Speak-up climate and respect for professional expertise

Two psychological safety dimensions were particularly influential: speak-up climate and respect for professional expertise. Effective internal communication enhances employee motivation and job satisfaction [13]. Prior studies have linked speak-up environments with improved patient safety culture and reduced turnover intention [14]. Our findings extend this evidence by demonstrating associations with broader healthcare workers’ perceptions of their organizational environment and perceived quality of care.

Recognition of professional expertise also appears critical. Modern healthcare increasingly relies on interdisciplinary collaboration, and diminished professional autonomy has been associated with increased turnover intention [15]. Environments that acknowledge and effectively utilize individual expertise may therefore support both staff satisfaction and workforce retention.

### Institutional initiatives and future directions

Leadership is widely recognized as a fundamental determinant of psychological safety. Leaders who encourage open communication, actively seek input from team members, and respond constructively to concerns help create an environment in which employees feel safe to speak up and contribute their expertise. Therefore, organizational efforts to improve psychological safety should not rely solely on individual employees but should also emphasize leadership development and supportive management practices.

In response to these findings, our institution has initiated leadership development programs and bottom-up improvement activities aimed at promoting participation in organizational decision-making and enhancing communication. Whether such initiatives improve psychological safety and successive retention intention and perceived quality of care requires longitudinal evaluation. Although psychological safety cannot be established rapidly, sustained organizational efforts may gradually foster workplaces in which employees feel comfortable expressing opinions and perceive their professional contributions as valued.

### Implications for healthcare organizations

Psychological safety should be considered a measurable organizational factor rather than solely a cultural concept. Interventions targeting communication climate and professional recognition may provide practical approaches for improving staff experience and organizational sustainability. Because staff experience and healthcare performance are closely interconnected, psychological safety may represent an important organizational factor worthy of future interventional investigation.

### Limitation

Several limitations should be acknowledged. This was a single-center cross-sectional study, limiting causal inference and generalizability. Because of the cross-sectional design, causal relationships between psychological safety and organizational outcomes cannot be established. All measures were based on self-reported questionnaires and may be subject to self-selection bias [16]. Although the overall evaluation was used as a global indicator of perceptions of their organizational environment, it was derived from a single-item measure. Although single-item global evaluations are commonly used in organizational surveys to capture overall impressions, they may not fully reflect the multidimensional nature of perceptions of their organizational environment. Future multicenter and longitudinal studies are needed to confirm causal relationships and evaluate whether interventions targeting psychological safety lead to sustained improvements in staff retention and patient-related outcomes. Participation was voluntary, and non-response bias cannot be excluded. To preserve complete anonymity, demographic information on non-responders and the overall workforce was not collected, precluding assessment of respondent representativeness. Therefore, our findings should be interpreted with appropriate caution. We performed multivariable analyses, but we cannot completely deny the presence of other potential residual confounders.

## Conclusions

Psychological safety was strongly associated with global organizational evaluation and key priority domains, including retention intention and perceived quality of care, among healthcare workers in a university hospital. In particular, environments that promote open communication and respect for professional expertise may represent essential components for improving staff satisfaction and organizational sustainability. Strengthening psychological safety may therefore serve as a practical strategy to enhance both workforce stability and healthcare quality.

## Supplementary Information

Below is the link to the electronic supplementary material.

Supplementary Material 1

Supplementary Material 2

## Data Availability

The datasets used and/or analyzed during the current study are available from the corresponding author upon reasonable request.
