# Supplementary material for: Associations of psychological safety and perceptions of organizational environment among healthcare workers: a cross-sectional study in a university hospital
Source: BMC Health Serv Res. 2026 Aug 7;26:1530. doi: 10.1186/s12913-026-15330-3 (PMC13737624; doi:10.1186/s12913-026-15330-3)
Supplement: Supplementary file 1 [file 12913_2026_15330_MOESM1_ESM.docx]

**Supplementary Table 1. Survey instruments and conceptual framework**

| **Survey item** | **Conceptual role** |
| --- | --- |
| ***Employee satisfaction*** |  |
| Overall evaluation (perceptions of their environment indicator) | Primary dependent variable |
| Retention intention | Priority domain / secondary dependent variable |
| Perceived quality of care | Priority domain / secondary dependent variable |
| Workplace atmosphere and relationships | Explanatory variable (independent variable) |
| Job satisfaction | Explanatory variable (independent variable) |
| Employment conditions | Explanatory variable (independent variable) |
| Working conditions | Explanatory variable (independent variable) |
| Learning and growth opportunities | Explanatory variable (independent variable) |
| Mental anxiety (reverse meaning) | Explanatory variable (independent variable) |
| Fair evaluation | Explanatory variable (independent variable) |
| Trust in supervisor | Explanatory variable (independent variable) |
| ***Psychological safety*** |  |
| Mistakes not held against members | Explanatory variable (independent variable) |
| Able to bring up problems | Explanatory variable (independent variable) |
| No rejection for being different | Explanatory variable (independent variable) |
| Safe to take interpersonal risks | Explanatory variable (independent variable) |
| Easy to ask colleagues for help | Explanatory variable (independent variable) |
| No deliberate undermining of others’ efforts | Explanatory variable (independent variable) |
| Skills and talents valued and utilized | Explanatory variable (independent variable) |

All items were rated using a 5-point Likert scale (1 = strongly disagree, 2 = disagree, 3 = neutral, 4 = agree, 5 = strongly agree).
