# Supplementary material for: Associations of psychological safety and perceptions of organizational environment among healthcare workers: a cross-sectional study in a university hospital
Source: BMC Health Serv Res. 2026 Aug 7;26:1530. doi: 10.1186/s12913-026-15330-3 (PMC13737624; doi:10.1186/s12913-026-15330-3)
Supplement: Supplementary file 2 [file 12913_2026_15330_MOESM2_ESM.docx]

**Supplementary Table 2. Questionnaire on Staff Satisfaction and Psychological Safety**

| **No.** | **Questions** |
| --- | --- |
| *Employee Satisfaction* | |
| 1 | You recommend Toyama University Hospital as a workplace. (Overall evaluation) |
| 2 | You think the workplace atmosphere and human relationships are good. (Workplace atmosphere and relationships) |
| 3 | You find your current job fulfilling. (Job satisfaction) |
| 4 | You are satisfied with your current employment conditions. (Employment conditions) |
| 5 | You are satisfied with your current working conditions. (Working conditions) |
| 6 | You think there are opportunities for learning and growth. (Learning and growth opportunities) |
| 7 | You think you can work without feeling mental anxiety. {Mental anxiety (reverse question)} |
| 8 | You think your work achievements and abilities are being evaluated fairly. (Fair evaluation) |
| 9 | You think you can trust your boss. (Trust in supervisor) |
| 10 | You want to continue working at this hospital. (Retention intention) |
| 11 | You recommend this hospital to someone you know. (Perceived quality of care) |
| *Psychological Safety* | |
| 12 | If you make a mistake on this team, it is not held against you. |
| 13 | Members of this team are able to bring up problems and tough issues. |
| 14 | People on this team do not reject others for being different. |
| 15 | It is safe to take a risk on this team. |
| 16 | It is not difficult to ask other members of this team for help. |
| 17 | No one on this team would deliberately act in a way that undermines your efforts. |
| 18 | Working with members of this team, your unique skills and talents are valued and utilized. |
